# TYRA-300, an FGFR3-selective inhibitor, promotes bone growth in two FGFR3-driven models of chondrodysplasia

**DOI:** 10.1172/jci.insight.189307

**Published:** 2025-04-03

**Authors:** Jacqueline H. Starrett, Clara Lemoine, Matthias Guillo, Chantal Fayad, Nabil Kaci, Melissa Neal, Emily A. Pettitt, Melissandre Pache, Qing Ye, My Chouinard, Eric L. Allen, Geneviève Baujat, Robert L. Hudkins, Michael B. Bober, Todd Harris, Ronald V. Swanson, Laurence Legeai-Mallet

**Affiliations:** 1 Tyra Biosciences, Carlsbad, California, USA.; 2 Université de Paris Cité, Imagine Institute, Laboratory of Molecular and Physiopathological Bases of Osteochondrodysplasia, INSERM UMR1163, Paris, France.; 3 Department of Genomic Medicine for Rare Diseases, French Reference Center for Constitutional Bone Diseases, Necker-Enfants Malades Hospital, Paris, France.

**Keywords:** Bone biology, Cell biology, Bone disease, Drug therapy, Mouse models

## Abstract

Achondroplasia (ACH) and hypochondroplasia (HCH), the two most common types of dwarfism, are each caused by *FGFR3* gain-of-function mutations that result in increased FGFR3 signaling, which disrupts chondrogenesis and osteogenesis, resulting in disproportionately shortened long bones. In this study, TYRA-300, a potent and selective FGFR3 inhibitor, was evaluated in 3 genetic contexts: wild-type mice, the *Fgfr3^Y367C/+^* mouse model of ACH, and the *Fgfr3^N534K/+^* mouse model of HCH. In each model, TYRA-300 treatment increased nasoanal length and tibia and femur length. In the two FGFR3-altered models, TYRA-300–induced growth partially restored the disproportionality of long bones. Histologic analysis of the growth plate in *Fgfr3^Y367C/+^* mice revealed that TYRA-300 mechanistically increased both proliferation and differentiation of chondrocytes. Importantly, children with ACH can experience medical complications due to foramen magnum stenosis, and TYRA-300 significantly improved the size and shape of the skull and foramen magnum in *Fgfr3^Y367C/+^* mice. Spinal stenosis is also a frequent complication, and TYRA-300 increased the lumbar vertebrae length and improved the shape of the intervertebral discs in both models. Taken together, these studies demonstrate that the selective FGFR3 inhibitor TYRA-300 led to a significant increase in bone growth in two independent FGFR3-driven preclinical models as well as in wild-type mice.

## Abstract 2

TYRA-300, an FGFR3-selective kinase inhibitor, increases bone growth in mouse models of achondroplasia and hypochondroplasia.

## Graphical abstract

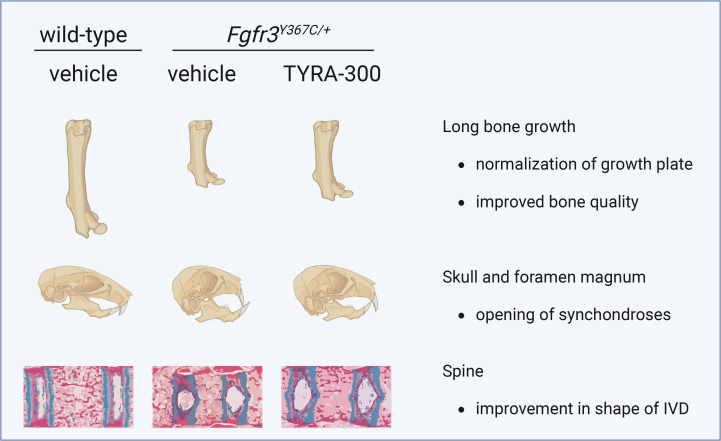

## Introduction

The FGF family of receptor tyrosine kinases is composed of 4 membrane-associated proteins, FGFR1, FGFR2, FGFR3, and FGFR4, that function to regulate cellular growth, differentiation, and homeostasis in numerous tissues (1–3). The FGF receptors (FGFRs) are composed of an extracellular domain, a transmembrane domain, and an intracellular tyrosine kinase domain. A fifth family member, FGFR5, consists of the extracellular and transmembrane domain but lacks the tyrosine kinase domain. Members of the FGF family of ligands interact with the extracellular domain to modulate activity of the intracellular kinase domain. Twenty-two FGF family members have been identified, 18 of which are secreted (1–3).

In the physis (or growth plate), FGFR3 is primarily expressed in proliferative zone chondrocytes (PZCs), with FGF2, FGF9, and FGF18 identified as the most important ligands (4). In nonpathologic conditions, activation of FGFR3 occurs when FGF ligands bind to the extracellular domain of the receptor in the presence of heparan sulfate proteoglycans. This promotes dimerization of FGFR3 monomers, which induces activation of the intracellular tyrosine kinase domain. Tyrosine kinase activation leads to subsequent downstream activation of the STAT1, ERK1/2, and p38 branches of the MAPK intracellular signaling pathways (1–3). Histologically, the physis consists of 3 principal layers of cells: the resting zone chondrocytes (RZCs), the PZCs, and the hypertrophic zone chondrocytes (HZCs). The RZCs serve as the progenitors that give rise to the PZCs. Oriented into cellular columns, the PZCs differentiate into HZCs. The HZCs produce an extracellular matrix that begins to mineralize, and ultimately, this calcified cartilage is replaced by bone. The rate of longitudinal bone growth is driven by the rate of proliferation and differentiation of the physeal chondrocytes (5–7).

The critical role of the FGF/FGFR3 axis in cartilage biology has been elucidated through human genetics. In 1994, one recurrent mutation in *FGFR3* was identified as causing achondroplasia (ACH) (8, 9). There are now 5 distinct *FGFR3*-related chondrodysplasias caused by specific gain-of-function mutations in *FGFR3* within this family, which range from the very severe and typically perinatal lethal forms of dwarfism (thanatophoric dysplasia type I and type II) to the most common ACH and severe ACH with acanthosis nigricans and developmental delay (SADDAN) and the milder short stature condition, hypochondroplasia (HCH) (6, 10). These spectrum conditions share clinical features of short-limbed dwarfism with either absolute or relative macrocephaly and midface retrusion. Although rare, autosomal dominant, heterozygous loss-of-function *FGFR3* mutations have also been described in humans with essential features of tall stature (>2 SD), camptodactyly, and hearing loss (11, 12).

The worldwide incidence of ACH is approximately 1 in 25,000 live births. Approximately 99% of cases of ACH are caused by either c.1138G>A (~98%) or c.1138G>C (~1%) *FGFR3* mutations (13). Both of these mutations lead to the same amino acid substitution, p.Gly380Arg, and are 100% penetrant (6, 14). There are distinct and recognizable features of ACH, which include disproportionately shortened limbs (relative to the trunk) with rhizomelia, decreased elbow extension, brachydactyly with trident configuration, macrocephaly with frontal bossing, and midface retrusion (6, 14, 15). HCH is a relatively common chondrodysplasia that may approach the prevalence of ACH, but no studies attempting to determine the prevalence of HCH have been published to our knowledge (16). Approximately 70% of cases of HCH are caused by either c.1620C>A or c.1620C>G mutations. Both of these mutations lead to the same amino acid substitution, p.Asn540Lys (17–19). The skeletal features of HCH are similar to, but less pronounced than, those of ACH and include disproportionate short stature with rhizomelia and absolute or relative macrocephaly (16). Children with HCH are less likely to experience the medical complications experienced by some children with ACH, but intellectual disability and epilepsy may be more common (16).

A variety of mouse models have been generated using transgenic, Cre/loxP, and other knockin approaches to recapitulate the human phenotypes by inserting mutations associated with FGFR3-related chondrodysplasias (20–22). When the ACH-associated mutations were inserted into mouse models, the FGFR3-altered mice exhibited a dwarfism characterized by short limbs, a small rib cage, craniofacial abnormalities, and a reduced foramen magnum. *Fgfr3*-altered mouse models (20) and fetal human growth plate cartilage analyses (23) demonstrated that gain-of-function mutations in *Fgfr3* and *FGFR3* cause disruption of the PZCs and HZCs within the physes. Conversely, loss-of-function *Fgfr3^–/–^* mice exhibit curved and elongated long bones, kyphosis, scoliosis, crooked tails, vertebrae abnormalities, and hearing loss (24).

The only therapy currently approved in the United States, European Union, and other limited countries to improve growth in children with ACH is vosoritide. Vosoritide is a C-natriuretic peptide (CNP) analog that is a once daily subcutaneous injection (25). In a pivotal study, children with ACH aged 5–12 years, who received vosoritide had an increase from baseline of average linear growth velocity of 1.57 cm/year (95% CI, 1.22–1.97 cm/year) greater than those given a placebo (25). While an important advance, long-term effects on ACH-associated comorbidities are not yet known. Other therapeutic approaches have aimed to target FGFR3 or its ligands, including an aptamer targeting FGF2 (26), a soluble FGFR3 decoy receptor (recifercept) that has been discontinued (27), and anti-FGFR3 antibodies. Most recently, small-molecule tyrosine kinase inhibitors (TKI) have been evaluated as an approach to inhibit FGFR3 activity. Infigratinib, a pan-FGFR1/2/3 inhibitor, showed proof-of-concept activity in an FGFR3-driven murine model of dwarfism (28, 29) and subsequently entered clinical trials in children with ACH (30).

In the present study we evaluate the efficacy of TYRA-300, a potent FGFR3-selective small-molecule TKI, in wild-type mice and 2 mouse models of FGFR3-driven chondrodysplasia. TYRA-300, designed to avoid the toxicities associated with inhibition of FGFR1, FGFR2, and FGFR4 (31), may provide greater efficacy with a larger therapeutic window than nonisoform-selective FGFR inhibitors. Here, the pharmacokinetic profile of TYRA-300 and effects on growth were evaluated in wild-type mice. TYRA-300 previously demonstrated in vitro potency against the FGFR3 G380R and N540K mutant proteins found in ACH and HCH, respectively (31). Therefore, the efficacy of TYRA-300 was evaluated in vivo in the *Fgfr3^Y367C/+^* mouse model mimicking the phenotype of ACH as well as in the *Fgfr3^N534K/+^* mouse model of HCH, in which infigratinib previously showed activity. In this paper, we demonstrate the strong efficacy of TYRA-300 on the physis and foramen magnum in these 2 Fgfr3-related mouse models.

## Results

### TYRA-300 increases growth velocity and long bone growth in wild-type mice.

**Figure 1:**
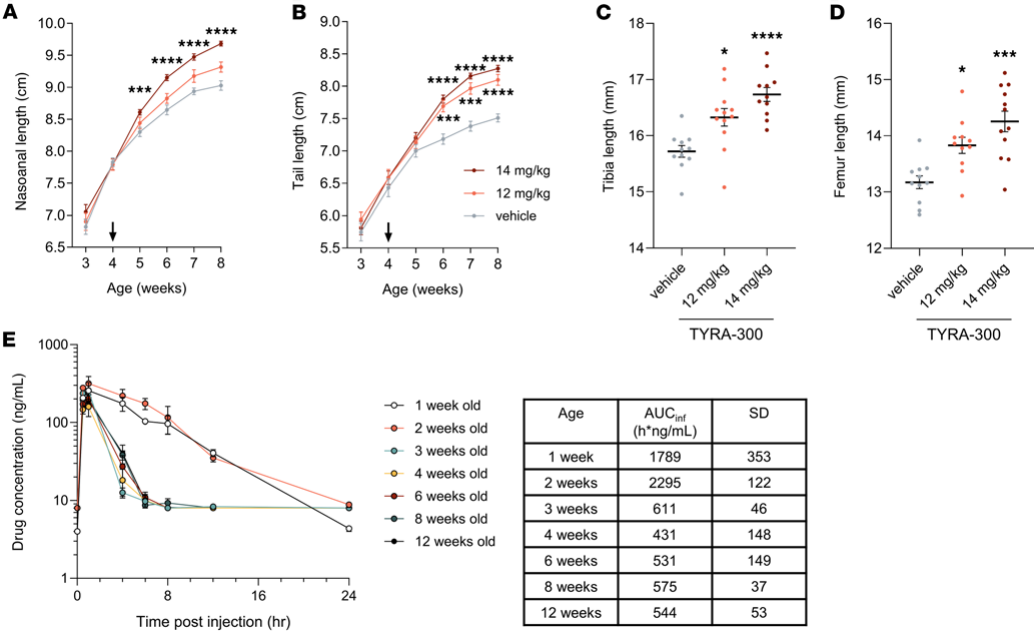
TYRA-300 demonstrates dose-dependent increases in growth velocity and long bones in a wild-type mouse model. (**A**) Nasoanal length and (**B**) tail length of female C57BL/6 mice from 3 to 8 weeks of age while receiving daily oral treatment with vehicle (*n* = 11), 12 mg/kg TYRA-300 (*n* = 12), or 14 mg/kg TYRA-300 (*n* = 12) from 4 to 8 weeks of age. Arrows indicate the start of treatment. Significance was assessed using a Mann-Whitney *U* test at each time point. (**C**) Tibia and (**D**) femur lengths measured by calipers on the final day of treatment (8 weeks of age). Significance for TYRA-300–treated groups versus the vehicle-treated group was assessed using a Kruskal-Wallis test. (**E**) Pharmacokinetic profile for female and male C57BL/6J mice ranging from 1 week old to 12 weeks old after a single subcutaneous dose of TYRA-300 (1.2 mg/kg). The mean AUC_inf_ (ng × hr/mL) for each age group is shown. For 1- and 2-week-old mice, data points represent *n* = 6–8 mice per time point. For 3- to 12-week-old mice, data points represent *n* = 4 mice. The lower limit of quantification was 4 ng/mL for the 1-week-old samples and 8 ng/mL for all other samples. Data in graphs represent mean ± SEM. *P < 0.05, ***P < 0.001, ****P < 0.0001.

To determine the in vivo effect of FGFR3 inhibition under normal physiologic conditions, TYRA-300 was tested in a growth velocity assay in wild-type mice (32). In this study, wild-type C57BL/6J mice were dosed orally with TYRA-300 once daily from 4 to 8 weeks of age. Previous work has demonstrated efficacy with TYRA-300 up to 18 mg/kg in oncology models (31). Based on the doses evaluated for infigratinib in oncology and ACH, a lower dose was thought to be more appropriate for long-term treatment of pediatric growth–related conditions. We therefore initially explored doses in the 8 to 14 mg/kg range in this model. Compared with the vehicle-treated group, there was a statistically significant increase in nasoanal length after treatment with 14 mg/kg TYRA-300 once daily (+7.3%, Figure 1A). Tail length and tibia and femur length also increased significantly and dose-dependently with both 12 mg/kg and 14 mg/kg TYRA-300 compared with vehicle treatment after 4 weeks of treatment (Figure 1, B–D). Specifically, after treatment with 12 mg/kg TYRA-300, the tibias were on average 3.9% longer than the tibias of mice that received treatment with vehicle, and the femurs were on average 5.0% longer after treatment; additionally, with 14 mg/kg TYRA-300, the tibias were 6.4% longer and the femurs were 8.2% longer. There was no difference in body weight among the treatment groups (data not shown). In addition, significant increases in nasoanal length and tibia and femur length were observed after treatment with 8 mg/kg and 10 mg/kg TYRA-300 (data not shown; *P* < 0.05). These results indicate that inhibition of wild-type FGFR3 signaling can effectively enhance growth velocity in a dose-dependent manner. While this model does not recapitulate any genetic growth disorder it does represent an accessible and facile tool for preliminary screening of compounds or treatments relative to the genetically modified models.

### TYRA-300 pharmacokinetic profile in juvenile mice.

The pharmacokinetic profile of TYRA-300 in adult rats has been previously described (31). Because the FGFR3-driven skeletal dysplasia models are performed using neonatal mice, an experiment was designed to understand the pharmacokinetics of TYRA-300 at a relevant efficacious dose in male and female mice, ranging from 1 to 12 weeks of age. In this experiment, TYRA-300 plasma levels were measured following administration of a single 1.2 mg/kg subcutaneous dose. TYRA-300 demonstrated a favorable pharmacokinetic profile across all mouse ages (Figure 1E). In 1-week-old mice, the AUC versus time calculated using 0 to infinity (AUC_inf_) was 1,789 h × ng/mL, with a maximum observed concentration of 287 ng/mL. There was a similar AUC_inf_ in 2-week-old mice (2,295 h × ng/mL). In 3- through 12-week-old mice, the plasma exposure was relatively consistent, with an average AUC_inf_ of 538 h × ng/mL, about 3.8 times lower than in the average of 1- and 2-week-old mice. Cytochrome P450 enzymes are commonly responsible for the metabolism of TKIs. In mice, cytochrome P450s are not fully developed until the mice are at least 3 weeks of age (33), which may explain the higher level of TYRA-300 exposure in the younger mice.

### TYRA-300 increases bone length in the Fgfr3^Y367C/+^ mouse model, which mimics the phenotype of ACH.

**Figure 2:**
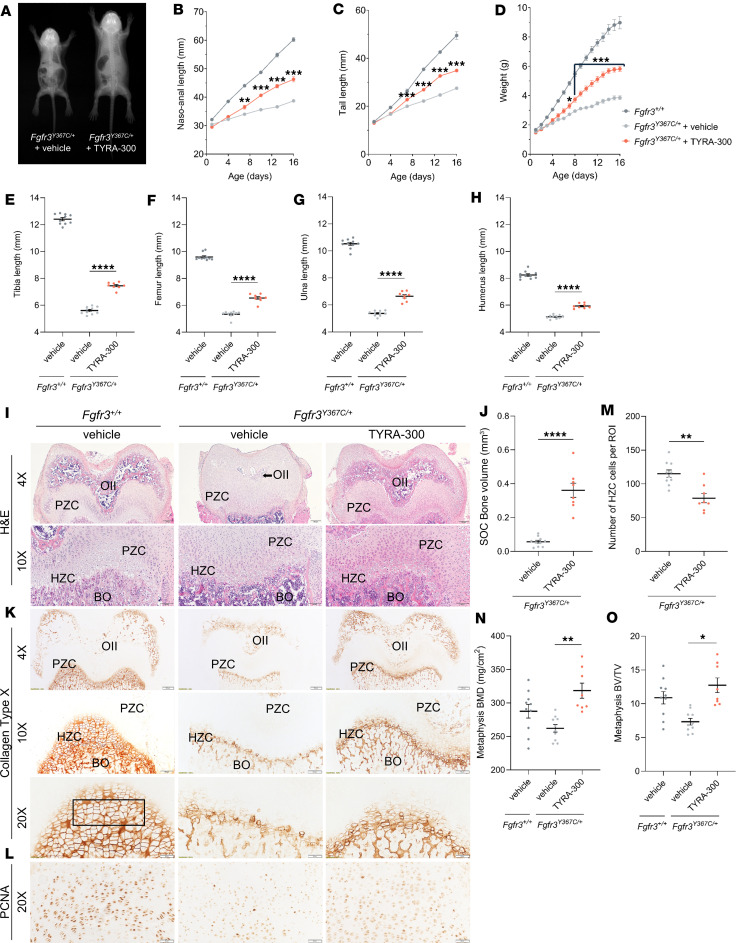
TYRA-300 increases long bone length through modulation of the growth plate in the *Fgfr3^Y367C/+^* mouse model, which mimics the phenotype of achondroplasia. (**A**) Representative whole-body radiographs of a vehicle-treated *Fgfr3^Y367C/+^* mouse on the left versus a TYRA-300–treated *Fgfr3^Y367C/+^* mouse on the right. Treatment consisted of daily subcutaneous injection of TYRA-300 at 1.2 mg/kg/d for 15 days starting at day 1 after birth. (**B**) Nasoanal length, (**C**) tail length, and (**D**) body weight of vehicle-treated wild-type (*Fgfr3^+/+^*) mice (*n* = 10), vehicle-treated *Fgfr3^Y367C/+^* mice (*n* = 10), and TYRA-300–treated *Fgfr3^Y367C/+^* mice (*n* = 8) from 1 to 16 days of age. Significance was assessed using a Mann Whitney *U* test at each time point. (**E**) Improvement in tibia, (**F**) femur, (**G**) ulna, and (**H**) humerus length. (**I**) Representative histological images of H&E-stained distal femurs (original magnification, ×4 [top]; ×10 [bottom]). (**J**) Quantification of bone volume of the SOC from μCT imaging. (**K**) Representative histological images of collagen type X (original magnification, ×4 [top], ×10 [middle], and ×20 [bottom]) and (**L**) PCNA (original magnification, ×20) staining of the distal femurs. (**M**) Number of HZC cells per region of interest (ROI), quantified from the same ROI within the chondro-osseous junction of the distal part of the right femur of each mouse, as denoted by the black box in **K** (original magnification, ×20 [950 × 342 pixels]). Scale bar: 200 μm (4× images); 100 μm (10× images); 50 μm (20× images). (**N**) Bone mineral density (BMD) and (**O**) bone volume to tissue volume (BV/TV) quantified from μCT imaging of the femurs. PCNA, proliferating cell nuclear antigen; PZC, proliferating zone chondrocytes; HZC, hypertrophic zone chondrocytes; OII, secondary ossification center; BO, bone. Significance for the TYRA-300–treated group versus vehicle-treated group was assessed using a Kruskal-Wallis test. *P < 0.05, **P < 0.01, ***P < 0.001, ****P < 0.0001. The bracket in **D** denotes that each time point (day 8–16) had a significance value of *P* < 0.001. Data in graphs represent mean ± SEM.

TYRA-300 was studied in a *Tg-CMV^Cre/+^*/*Fgfr3^Y367C/+^* mouse model, which, while corresponding to the human FGFR3 Y373C mutant responsible for thanatophoric dysplasia, type I, displays a phenotype similar to that of human ACH and has been previously used to test the preclinical efficacy of other therapies in this context (21, 28, 29, 34–36). In this study, *Fgfr3^Y367C/+^* mice were administered TYRA-300 daily by subcutaneous injection at a dose of 1.2 mg/kg for 15 days beginning at 1 day of age to evaluate the effects on growth, bone length, and key skeletal parameters, compared with those of vehicle-treated *Fgfr3^Y367C/+^* mice. Results were also compared with those from vehicle-treated *Fgfr3^+/+^* (wild-type) mice. Representative x-ray images at the end of the study clearly demonstrate an overall increase in the size of the *Fgfr3^Y367C/+^* mice after treatment with TYRA-300 compared with vehicle treatment (Figure 2A). TYRA-300 progressively increased nasoanal length in the mutant mice by 17.88% and tail length by 25.10% compared with the vehicle-treated mutant mice, as seen in the growth curves over the course of treatment (Figure 2, B and C). TYRA-300–treated mice also demonstrated a progressive increase in body weight (Figure 2D). At the end of treatment, TYRA-300–treated mice showed a 52.9% increase in body weight compared with vehicle-treated mice, suggesting an overall beneficial effect on the *Fgfr3^Y367C/+^* mice. TYRA-300 treatment resulted in statistically significant increases in the length of the tibia (+33.01%, Figure 2E), femur (+22.55%, Figure 2F), ulna (+23.51%, Figure 2G), and humerus (+15.52%, Figure 2H) compared with vehicle-treated mice. In *Fgfr3^Y367C/+^* mice, the tibia and ulna were shortened to a greater extent than the femur and humerus, resulting in disproportionate long bones. TYRA-300 treatment resulted in a greater effect on the tibia and ulna than the femur and humerus, demonstrating that proportionality was partially restored.

### TYRA-300 restored the architecture of the growth plate through increased proliferation and differentiation of chondrocytes.

To understand the effects of TYRA-300 treatment on the growth plate cartilage and chondrocytes and the mechanism through which TYRA-300 increases bone growth, histological analysis of the femurs of *Fgfr3^Y367C/+^* mice was performed. H&E images of the distal femur showed an increase in the overall size of the femoral distal epiphysis in TYRA-300–treated *Fgfr3^Y367C/+^* mice compared with vehicle-treated *Fgfr3^Y367C/+^* mice (Figure 2I). The secondary ossification center (SOC) in the distal epiphysis was larger in TYRA-300–treated mice than that observed in vehicle-treated *Fgfr3^Y367C/+^* mice, suggesting that SOC formation was promoted by TYRA-300 treatment. Micro-CT (μCT) analyses confirm the histological data showing a significant increase of the bone volume of the SOC in TYRA-300–treated *Fgfr3^Y367C/+^* mice (Figure 2J and Supplemental Figure 1, A and B; supplemental material available online with this article; https://doi.org/10.1172/jci.insight.189307DS1). Treatment also facilitated angiogenesis, deposition of bone matrix, and resorption of the cartilage. In addition, the overall structure of the growth plate was more organized after treatment with TYRA-300. Higher magnification (Figure 2I) showed improved organization of the PZCs in TYRA-300–treated mice, whereas it was disorganized in the vehicle-treated mice. The organization of the hypertrophic zone, with HZCs organized in columns, appeared similar in TYRA-300–treated *Fgfr3^Y367C/+^* mice and *Fgfr3^+/+^* mice. Within the hypertrophic zone of TYRA-300–treated mice, the HZCs were larger than in vehicle-treated mice.

Collagen type X is a specific marker of terminally differentiated HZCs. Consistent with the H&E stain, collagen type X immunostaining of the distal femoral growth plate revealed a change in the pattern of staining around the region of the SOC to reflect its increased development in TYRA-300–treated mice compared with vehicle-treated *Fgfr3^Y367C/+^* mice (Figure 2K). This suggests that TYRA-300 treatment resulted in an increase in the differentiation of chondrocytes and improved organization of the growth plate.

To assess the effects of TYRA-300 on proliferation of chondrocytes in the distal femoral growth plate, immunostaining for proliferating cell nuclear antigen (PCNA) was performed. PCNA is a specific marker of cell proliferation. TYRA-300 treatment modified the expression of PCNA by creating a more well-defined positive zone within the growth plate, indicating an increase in PZCs (Figure 2L).

To further analyze the changes in the hypertrophic zone after treatment with TYRA-300, the number of HZCs within the same region of interest (ROI) for each image was quantified. Interestingly, there was a significant decrease in the number of HZCs within the ROI in the TYRA-300–treated *Fgfr3^Y367C/+^* mice compared with vehicle-treated mice (Figure 2M), indicating that the size of the HZCs was increased. Therefore, TYRA-300 treatment increased bone growth by increasing both proliferation and differentiation of chondrocytes, resulting in an overall growth plate structure that is more similar to a wild-type growth plate.

Finally, measurements of trabecular bone quality were performed using μCT imaging of the femurs (Supplemental Figure 2). Analysis of the femoral metaphyses revealed that treatment with TYRA-300 increased the bone mineral density (BMD, +21.4%, Figure 2N) and bone volume–to–tissue volume ratio (BV/TV, +73.3%, Figure 2O) in *Fgfr3^Y367C/+^* mice, suggesting that TYRA-300 improved bone quality and strength in this model.

### TYRA-300 improved the size and shape of the skull and foramen magnum.

**Figure 3:**
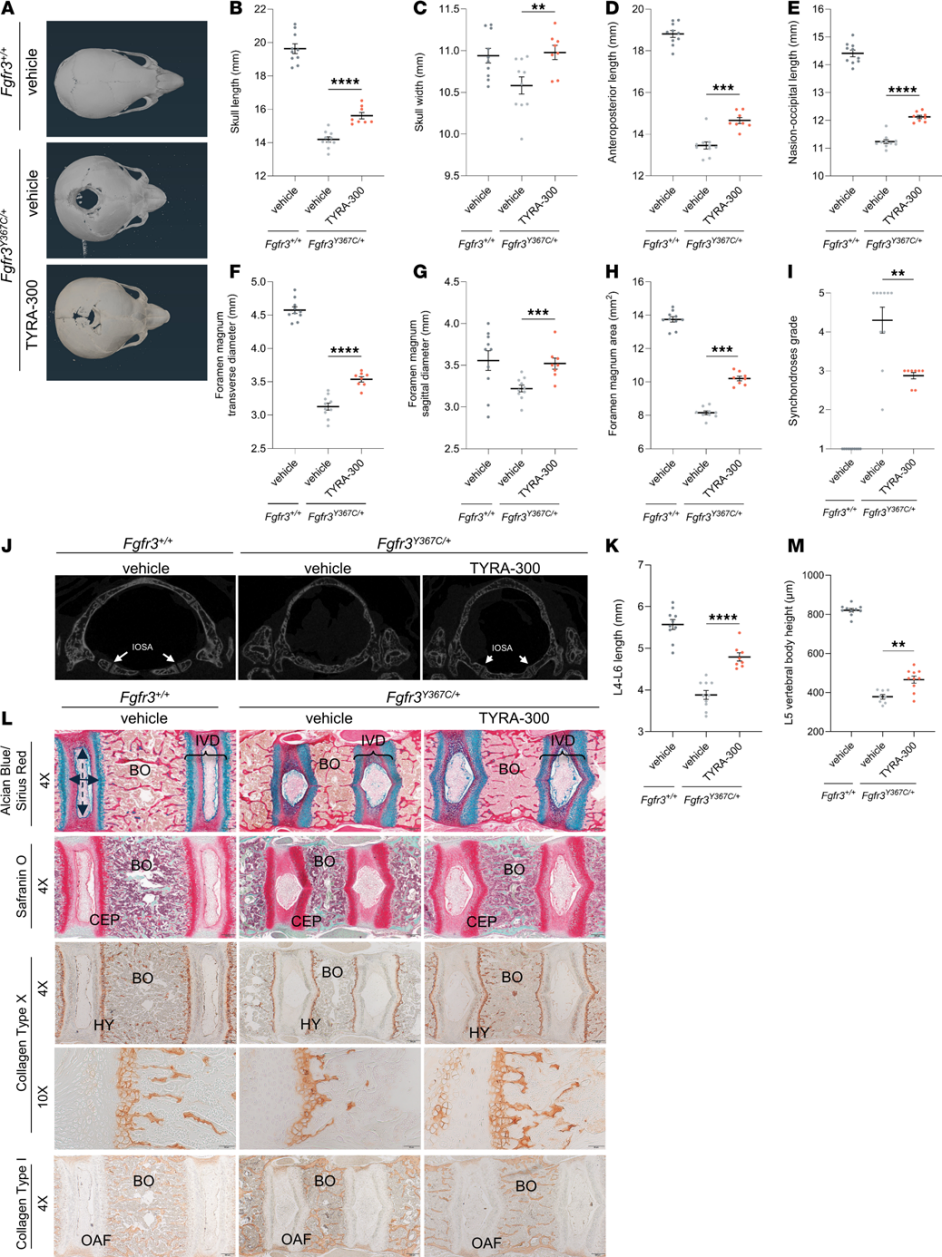
TYRA-300 improved the size and shape of the axial skeleton in *Fgfr3^Y367C/+^* mice. (**A**) Representative μCT images of the skull. (**B**) Skull length, (**C**) width, (**D**) anteroposterior length, and (**E**) nasio-occipital length of vehicle-treated wild-type (*Fgfr3^+/+^*) mice (*n* = 10), vehicle-treated *Fgfr3^Y367C/+^* mice (*n* = 10), and TYRA-300–treated (1.2 mg/kg/d s.c.) *Fgfr3^Y367C/+^* mice (*n* = 8) from 1 to 16 days of age. (**F**) Improvement in foramen magnum transverse diameter, (**G**) sagittal diameter, (**H**) area, and (**I**) grade of synchondroses. (**J**) Representative μCT images of the foramen magnum from each group after treatment. Synchondroses were graded using the following key: I, border of synchondroses were completely separated; II, clear separation of synchondroses with some areas suspicious for bone bridging; III, synchondroses showing bony bridge between 2 borders; IV, completely fused synchondroses with remnants of margin (cartilage); and V, completely fused synchondroses. IOSA, intraoccipital synchondrosis anterior. (**K**) Length of the L4–L6 lumbar vertebrae, as measured by calipers on the final day of the study. (**L**) Representative histological images of Alcian blue with Sirius red, Safranin O, collagen type X, and collagen type I staining of the L5 lumbar vertebrae after treatment (original magnification, ×4 [first, second, third, and last row], ×10 [second-to-last row]). The solid arrow line indicates height of nucleus pulpous, and the dashed arrow line indicates width of nucleus pulposus. Scale bar: 200 μm (4× images); 100 μm (10× images). (**M**) Quantification of the height of the L5 vertebral body from the center point of each Alcian blue/Sirius red image. IVD, intervertebral disc; BO, bone; CEP, cartilage end plate; HY, hypertrophic chondrocyte; OAF, outer annulus fibrosus. Significance was assessed using a Kruskal Wallis test. **P < 0.01, ***P < 0.001, ****P < 0.0001. Data in graphs represent mean ± SEM.

Another hallmark of ACH that is observed in *Fgfr3^Y367C/+^* mice is change in the size and shape of the skull and foramen magnum (28). The size of the foramen magnum is of particular clinical importance, as its reduced size can cause compression of the brain stem and spinal cord, resulting in significant morbidity and mortality in infants with ACH (37–40). To study the effects of TYRA-300 on the skull and foramen magnum in *Fgfr3^Y367C/+^* mice, macroscopic analyses were performed and μCT images were taken of the mice at the end of study. Changes in the shape of the skull are visible on μCT images in TYRA-300–treated *Fgfr3^Y367C/+^* mice compared with vehicle-treated *Fgfr3^Y367C/+^* mice (Figure 3A). Macroscopic analyses revealed that TYRA-300 treatment increased the length (+10.08%) and width (+3.74%) of the skull in *Fgfr3^Y367C/+^* mice compared with that in vehicle-treated *Fgfr3^Y367C/+^* mice (Figure 3, B and C). These changes in dimensions resulted in the overall shape of the skull being more similar to the shape of a wild-type mouse skull, with the width being similar to the width in wild-type mice as well (Figure 3A). The skull was further analyzed using the μCT images, which enabled measurement of the anteroposterior length and nasio-occipital length of the skull. TYRA-300 treatment significantly increased both the anteroposterior length (+8.92%, Figure 3D) and the nasio-occipital length (+7.87%, Figure 3E) in *Fgfr3^Y367C/+^* mice compared with vehicle-treated mice.

Macroscopic analyses also demonstrated that TYRA-300 treatment resulted in an increase in the transverse diameter (+13.05%, Figure 3F) and sagittal diameter (+9.28%, Figure 3G) of the foramen magnum. As with the skull, these changes resulted in the shape of the foramen magnum being more similar to the shape in a wild-type mouse. μCT images also enabled quantification of the area of the foramen magnum. TYRA-300 increased the area of the foramen magnum in *Fgfr3^Y367C/+^* mice by 25.17% compared with that in vehicle-treated mice (Figure 3H). The μCT images also indicate that treatment with TYRA-300 modified the synchondroses at the base of the skull (Figure 3, I and J). The intraoccipital synchondrosis anterior of each mouse was scored with a grade of I–V, with grade I indicating that the borders of the synchondroses were completely separated and grade V indicating that the synchondroses were completely fused (41). TYRA-300 treatment significantly improved the grade of the synchondroses, resulting in partially open synchondroses with grades of II or III in all treated mice (Figure 3I). This indicates that TYRA-300 might prevent the premature fusion of the synchondroses observed in ACH. Overall, the improvement in the size and shape of the foramen magnum with TYRA-300 has important clinical implications for foramen magnum stenosis.

### TYRA-300 improved the architecture of the lumbar vertebrae and shape of the intervertebral discs.

Individuals with ACH frequently experience back pain and may undergo multiple spinal surgeries throughout their lives (40). Kyphosis and scoliosis are common with ACH and are also observed in the *Fgfr3^Y367C/+^* mouse model (40), and Fgfr3 is expressed in the intervertebral discs of *Fgfr3^Y367C/+^* mice (28). To determine how TYRA-300 affects the vertebrae and intervertebral discs, the spine was analyzed in the *Fgfr3^Y367C/+^* mice treated with TYRA-300 or vehicle. The L4–L6 lumbar vertebrae segment length was increased (+23.49%) in TYRA-300–treated *Fgfr3^Y367C/+^* mice compared with vehicle-treated mice (Figure 3K).

The lumbar vertebrae were analyzed by immunohistochemical staining to further understand and visualize changes within the spine. These images revealed that TYRA-300 increased the height of the vertebral bodies in *Fgfr3^Y367C/+^* mice compared with vehicle-treated mice (Figure 3, L and M), a potential mechanism underlying the increase in spinal length. The bony trabeculae of TYRA-300–treated *Fgfr3^Y367C/+^* mice appeared similar to the bony trabeculae in wild-type mice. Surprisingly, TYRA-300 also modified the shape of the intervertebral disc — in particular, by decreasing the height and increasing the lateral width of the nucleus pulposus, resulting in a shape more similar to the shape observed in wild-type mice (Figure 3L). There was a slight change in collagen type X staining present in the lumbar vertebrae after treatment with TYRA-300 (Figure 3L). Collagen type I is expressed in the bone as well as outer annulus fibrosus, and its staining pattern suggests that the architecture of the bony trabeculae after TYRA-300 treatment was similar to that observed in the wild-type mouse, with thin and numerous trabeculae (Figure 3L). These data suggest that TYRA-300 may benefit both the bone architecture and the intervertebral disc abnormalities of the lumbar vertebrae.

### TYRA-300 increased bone length in an Fgfr3^N534K/+^ mouse model of HCH.

**Figure 4:**
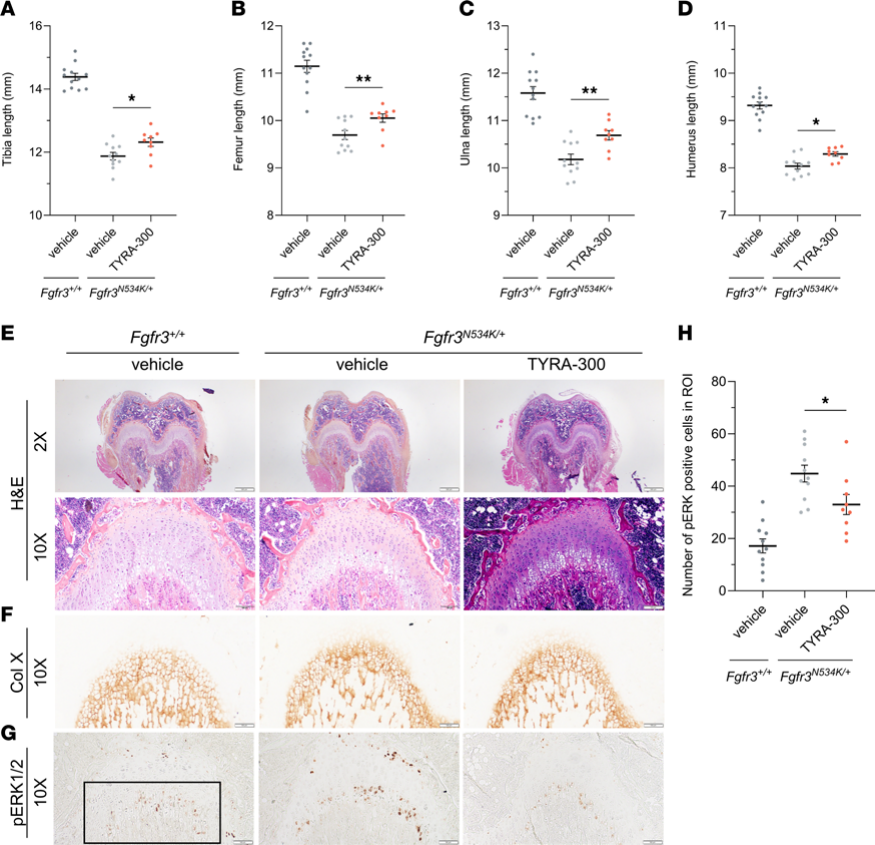
TYRA-300 increased bone length and reduced FGFR3 signaling within the growth plate in a *Fgfr3^N534K/+^* mouse model of hypochondroplasia. (**A**–**D**) Length of (**A**) tibia, (**B**) femur, (**C**) ulna, and (**D**) humerus in vehicle-treated wild-type (*Fgfr3^+/+^*) mice (*n* = 12), vehicle-treated *Fgfr3^N534K/+^* mice (*n* = 11), and TYRA-300–treated *Fgfr3^N534K/+^* mice (*n* = 9) after once daily treatment from 3 to 24 days of age. (**E**) Representative histological images of H&E (original magnification, ×2 [top], ×10 [bottom]), (**F**) collagen type X (original magnification, ×10), (**G**) and pERK1/2 staining of the distal femur (original magnification, ×10). (**H**) Number of pERK1/2^+^ cells per region of interest (ROI), quantified from the same ROI within the chondro-osseus junction of the right distal femur of each mouse, as designated by the black box in **G** (1,306 × 587 pixels). Scale bar: 500 μm (2× images); 100 μm (10× images). Col X, collagen type X. Significance was assessed using a Kruskal Wallis test. *P < 0.05, **P < 0.01. Data in graphs represent mean ± SEM.

To explore the effectiveness of TYRA-300 in an additional model of FGFR3-related chondrodysplasia, TYRA-300 was evaluated in a *Tg-CMV^Cre/+^/Fgfr3^N534K/+^* mouse model of HCH (42). This model is driven by the *Fgfr3* N534K mutation, which corresponds to the human *FGFR3* N540K mutation (9, 43). This model closely recapitulates the skeletal phenotype of HCH in humans, with a milder disproportionate short stature phenotype (42). A higher dose of TYRA-300 was explored in this model due to the lower inhibition of FGFR3 N540K relative to that in wild-type and G380R mutant proteins, as described previously (31). In preliminary dose exploration, a 2.4 mg/kg dose was initially evaluated, and phenotypic signs consistent with the *Fgfr3^–/–^* model were observed (data not shown) (24). Because the goal of treatment with TYRA-300 is to restore Fgfr3 signaling levels, *Fgfr3^N534K/+^* mice were administered a lower dose of 1.8 mg/kg TYRA-300 daily for the full study. Mice were treated for 21 days, from 3 to 24 days of age. Treatment was started at an older age in the HCH model than in the ACH model, as children with HCH are often diagnosed later than children with ACH. Treatment with TYRA-300 resulted in statistically significant increases in the appendicular skeleton, including the femur (+3.70%), tibia (+3.75%), ulna (+5.03%), and humerus (+3.22%), compared with vehicle-treated *Fgfr3^N534K/+^* mice (Figure 4, A–D). These data demonstrate that TYRA-300 was effective in increasing bone lengths in the *Fgfr3* N534K–driven mouse model of HCH.

To confirm the mechanistic effect of TYRA-300 within the growth plate of treated *Fgfr3^N534K/+^* mice, immunohistochemical analyses were performed on the distal femurs. In contrast to the ACH model, a larger SOC is visible in the femoral epiphysis of HCH mice (42), and the effect of TYRA-300 on SOC formation is not obvious. H&E and collagen type X images did not demonstrate significant changes in the overall architecture of the growth plate (Figure 4, E and F). It is well established that the MAPK pathway is the canonical FGFR3 downstream signaling pathway. Here, we confirmed that this pathway is still activated at 24 days of age in the HCH growth plate through immunohistochemical staining of phosphorylated ERK1/2 (pERK), which was positive in hypertrophic chondrocytes (Figure 4G). Quantification revealed a significant decrease in pERK^+^ cells after treatment with TYRA-300 compared with treatment with vehicle (Figure 4, G and H). This indicates that TYRA-300 treatment modulates FGFR3 signaling within the growth plate in the context of the *Fgfr3* N534K mutation.

### TYRA-300 increased growth of the axial skeleton in Fgfr3^N534K/+^ mice.

**Figure 5:**
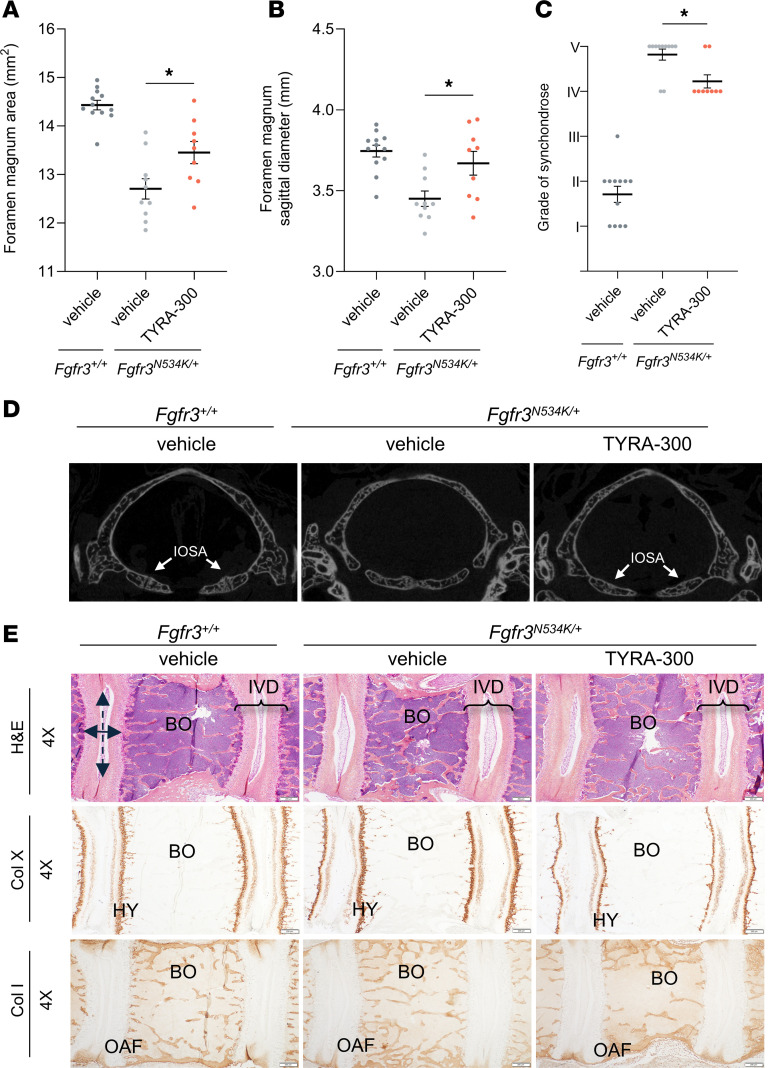
TYRA-300 increased the size of the foramen magnum and modified the shape of the intervertebral discs in *Fgfr3^N534K/+^* mice. (**A**) Area and (**B**) sagittal diameter of the foramen magnum of vehicle-treated wild-type (*Fgfr3^+/+^*) mice (*n* = 12), vehicle-treated *Fgfr3^N534K/+^* mice (*n* = 11), and TYRA-300–treated *Fgfr3^N534K/+^* mice (*n* = 9) after once daily treatment from 3 to 24 days of age. (**C**) Improvement in the grade of synchondroses after treatment. (**D**) Representative μCT images of the foramen magnum from each group. Synchondroses were graded using the following key: I, border of synchondroses were completely separated; II, clear separation of synchondroses with some areas suspicious for bone bridging; III, synchondroses showing bony bridge between 2 borders; IV, completely fused synchondroses with remnants of margin (cartilage); and V, completely fused synchondroses. IOSA, intraoccipital synchondrosis anterior. (**E**) Representative histological images of staining of the L5 lumbar vertebrae after 21 days of treatment: H&E (original magnification, ×4), collagen type X (original magnification, ×4), and collagen type I (original magnification, ×4). Scale bar: 200 μm. BO, bone; HY, hypertrophic chondrocyte; OAF, outer annulus fibrosus; Col X, collagen type X; Col I, collagen type I. Significance was assessed using a Kruskal Wallis test. *P < 0.05. Data in graphs represent mean ± SEM.

Finally, to confirm the effects of TYRA-300 on the axial skeleton in *Fgfr3^N534K/+^* mice, μCT imaging of the foramen magnum was performed at the end of treatment. Images revealed that TYRA-300 significantly increased the area of the foramen magnum (+7.51%, Figure 5A) as well as the sagittal diameter (+6.35%, Figure 5B) compared with vehicle treatment in *Fgfr3^N534K/+^* mice. There was not a statistically significant change in the transverse diameter (data not shown). Grading of the synchondroses also demonstrated a reduction from grade V (completely fused) to grade IV (fused with remnants of cartilage margin) after treatment with TYRA-300 in most mice (Figure 5C), as seen in the μCT images (Figure 5D).

We also analyzed the lumbar vertebrae and intervertebral disc (Figure 5E). As previously observed in *Fgfr3^Y367C/+^* mice, TYRA-300 slightly modified the shape of the intervertebral disc, in particular the nucleus pulposus. As observed in the femoral growth plate (Figure 4F), collagen type X immunolabeling revealed no obvious change in the collagen type X^+^ zone in the cartilage end plate, and there were also no obvious changes in collagen type I. Here, we can therefore conclude that TYRA-300 improved intervertebral disc abnormalities of the lumbar vertebrae of HCH mice.

## Discussion

Currently vosoritide is the only approved therapy in the United States, European Union, and limited other countries for increasing growth in children with ACH, except in Japan where human growth hormone is also approved (44). Vosoritide is an analog of CNP that acts downstream of FGFR3 and requires daily subcutaneous injections in pediatric patients over multiple years. While vosoritide has demonstrated promising improvement in annualized growth velocity, there remains an unmet need for an oral therapy that directly targets FGFR3 — the genetic driver of ACH and HCH — and provides meaningful clinical benefit for the medical complications associated with ACH. Infigratinib, a pan-FGFR1/2/3 inhibitor, is currently undergoing clinical trials as an oral therapy (30). In the present study, we evaluated the efficacy of TYRA-300, an FGFR3 isoform–selective inhibitor that may potentially provide a larger therapeutic window (31), in wild-type mice and 2 mouse models of FGFR3-driven chondrodysplasia.

Analogs of CNP including vosoritide have previously been evaluated in wild-type animals using different treatment regimens or mouse strains; they have also demonstrated increases in growth velocity and long bone length (32, 45, 46). Both vosoritide and infigratinib have also been evaluated in the *Fgfr3^Y367C/+^* mouse model of FGFR3-driven chondrodysplasia (28, 29, 34). In *Fgfr3^Y367C/+^* mice, when dosed starting at 7 days of age and continuing for 10 days, infigratinib (2 mg/kg) resulted in more bone growth than vosoritide (800 μg/kg) (28, 34). In the present study, TYRA-300 (1.2 mg/kg) resulted in more bone growth than low-dose infigratinib (0.5 mg/kg) when dosed for 15 days starting at 1 day of age (28, 29). TYRA-300 treatment resulted in significant growth of each of the long bones in both the *Fgfr3^Y367C/+^* and *Fgfr3^N534K/+^* mouse models. The values appear smaller in the HCH model than ACH model owing to the more moderate phenotype and smaller window of growth in the HCH model. However, TYRA-300 resulted in a similar percentage of normalization toward wild-type in both models (average of 25% normalization of long bone length toward wild-type in the HCH model, versus 26% in the ACH model). Together, these preclinical studies suggest that a direct FGFR3-targeted approach may be the most effective in the context of an activating mutation in FGFR3.

Beyond the functional challenges that present with disproportionate short stature (47), there are multiple comorbidities associated with ACH that can require invasive treatment. The CLARITY study, which is the largest ACH natural history study to date, studied 1,374 individuals with ACH over a 6-decade period and found that 79.6% of individuals underwent a total of 4,552 ACH-related surgical procedures — an average of 4.2 procedures per person who had a surgery (15). The most common surgical procedures were ear, nose, and throat operations related to abnormal endochondral bone formation in the skull base and midface, orthopedic surgery for lower extremity realignment, decompression of the foramen magnum, and surgical decompression due to spinal stenosis (15, 48–50). The goal of development of TYRA-300 is to address these types of serious medical complications in addition to functional challenges owing to disproportionate short stature. Therefore, the effect of TYRA-300 on the foramen magnum and spine was specifically analyzed in the present study.

Infants and children up to 5 years old with ACH have a 50-fold increase in mortality due to critical foramen magnum stenosis, leading to compression of the brain stem and upper cervical spinal cord (37, 39). Foramen magnum stenosis also predisposes infants with ACH to severe injury such as hemiparesis (51) and requires decompression surgery in approximately 20% of infants (50, 52). Importantly, TYRA-300 treatment increased the area of the foramen magnum in *Fgfr3^Y367C/+^* mice by 25.17% compared with vehicle-treated mice. It is thought that the cause for the changes in the foramen magnum in ACH is due to premature closure of the synchondroses of the skull base in addition to the defects in endochondral bone formation (53), in turn a direct result of overactive Fgfr3 signaling (54). Indeed, we observed premature closure of the intraoccipital synchondroses in the vehicle-treated *Fgfr3^Y367C/+^* mice, which was rescued with TYRA-300 treatment. Therefore, TYRA-300 may help prevent premature closure of the skull base synchondroses in addition to promoting endochondral bone growth. A similar effect was also observed with infigratinib in the *Fgfr3^Y367C/+^* mouse model (28). Given that infants with ACH are born with a significantly smaller foramen magnum than average and that synchondroses can close prematurely, there may be the potential to have a greater impact when treatment is started earlier in life or even prenatally. Indeed, a recent study in the *Fgfr3^G374R^* mouse model of ACH demonstrated that infigratinib treatment initiated 1 day after birth did not rescue premature fusion of the intersphenoidal synchondrosis, which contributes to elongation of the skull in the rostrocaudal direction (55).

Spinal stenosis is the single largest morbidity in people with ACH, with studies suggesting the need for spinal surgery in approximately 15% of individuals by 20 years of age and increasing throughout the life span to approximately 70% by 60 years of age (40, 49). Spinal stenosis is largely due to inhibited growth within the neurocentral synchondroses that lengthen the pedicles, resulting in decreased space available for the cord (56). Multiple other factors contribute to the need for surgical intervention in adult patients (40, 57, 58), several of which, including osteoarthritic changes and ligamentous and disc disease, have long been felt to be age-related and degenerative in nature (59). Data emerging from these preclinical models and other sources now point to intervertebral disc disease being a primary manifestation of ACH and other FGFR3-opathies. The nucleus pulposus from both the *Fgfr3^Y367C/+^* and *Fgfr3^N534K/+^* mouse models show disorganization with central thickening and lateral narrowing. Treatment with TYRA-300 improved the intervertebral disc shape in both models. FGFR3 can be visualized by immunohistochemical methods in human fetal intervertebral discs between 12- and 20-weeks’ gestation (60). Furthermore, there is an increased thickness of the intervertebral discs in infants with ACH, as assessed radiographically (61). Taken together, these data suggest that the abnormal intervertebral disc development present in the mouse models may also be present in human ACH and may respond to treatment with TYRA-300 if given within the appropriate window.

Type X collagen is a specific marker for terminally differentiated HZCs. Its degradation byproduct, collagen X marker (CXM), consists of the intact trimeric noncollagenous 1 domain of type X collagen and is measurable in serum and plasma. Blood levels of CXM correlate with height velocity in healthy children. In a study of children with ACH, CXM was significantly lower than in the general population (62), consistent with the lack of staining for type X collagen in the HZCs of the *Fgfr3^Y367C/+^* vehicle-treated mice. Treatment with TYRA-300 in the present study and previously with vosoritide and infigratinib resulted in a partial restoration of the type X collagen staining in the HZCs indicating improved HZC function. Elevation of serum CXM was similarly observed in clinical trial participants treated with vosoritide and infigratinib when doses became high enough to change annualized growth velocity. Thus, CXM may be an important human biomarker that corresponds to histological and functional improvement of growth plate physiology (63, 64). Increased growth velocity has also been reported in several pediatric oncology cases after treatment with pan-FGFR inhibitors (65–67). These children did not have genetically defined short stature conditions, yet still experienced annualized growth velocities above the expected range as a result of FGFR inhibition and, in some cases, experienced complications such as slipped capital femoral epiphyses attributed to the accelerated growth. These observations along with the ability of TYRA-300 to increase growth velocity in wild-type mice demonstrated here suggest that FGFR inhibition could promote bone growth in short stature conditions that are not directly related to FGFR3 genetic alterations. However, as with the use of growth hormone therapy, the risk-to-benefit ratio of a TKI therapy would require evaluation for these populations.

Overall, the current study demonstrates that TYRA-300 effectively promotes bone growth in mice in the context of both wild-type FGFR3 signaling and FGFR3 altered chondrodysplasias. TYRA-300 is the first FGFR3-selective small-molecule inhibitor to be evaluated in these models to our knowledge and may provide a greater therapeutic window than pan-FGFR inhibitors currently in development. Together, these data provide rationale for the clinical study of TYRA-300 in children with ACH, HCH, and potentially other FGFR3-related osteochondrodysplasias.

## Methods

### Sex as a biological variable.

The wild-type growth velocity study was performed exclusively in female mice, as previous studies demonstrated similar findings in both sexes (32). The pharmacokinetic study and studies in *Fgfr3^Y367C/+^* and *Fgfr3^N534K/+^* mice were performed in a mixture of male and female mice.

### Animals.

The ACH *Fgfr3^Y367C/+^* mouse model was previously described (21, 34). *Fgfr3^Y367C/+^* mice were generated by crossing CMV-Cre (C57BL/6J) mice with mice exhibiting germline transmission of the Y367C mutation corresponding to the human Y373C mutation. The HCH *Fgfr3^N534K/+^* mouse model was also previously described (42). *Fgfr3^N534K/+^* mice were generated by crossing CMV-Cre (C57BL/6J) mice with mice exhibiting germline transmission of the N534K mutation corresponding to the human N540K mutation.

### Pharmacokinetic study.

C57BL/6J (The Jackson Laboratory) mice were randomized to treatment groups according to body weight. Owing to the young age of some groups, a mixture of female and male mice was used. Depending on the age of mice, 3–8 mice per group were dosed with a single subcutaneous dose of TYRA-300 (1.2 mg/kg) and then blood was collected either serially at 3 different time points (if 4 weeks of age and older) or terminally at a single time point (if 1–3 weeks of age). Mouse plasma was harvested using lithium heparin tubes at 0.5, 1, 4, 6, 8, 12, and 24 hours after dosing. Compound concentration from plasma was detected using liquid chromatography with tandem mass spectrometry at Oakland Analytics.

### Wild-type mouse growth velocity study.

At 21 days of age, female C57BL/6J mice (The Jackson Laboratory) were subject to nasoanal length and nasotail length measurement while under anesthesia. Tail length was calculated by subtracting nasoanal length from nasotail length. Body weights were also recorded. One week later, at 28 days of age, nasoanal lengths and body weights were measured again and mice were randomized to treatment groups of 12 mice per group according to nasoanal length. TYRA-300 was dissolved in 30% hydroxypropyl-β-cyclodextrin for administration by oral gavage. Mice were dosed orally with TYRA-300 or vehicle control once daily (QD) for 4 weeks, and nasoanal lengths and body weights were monitored once weekly, along with daily clinical observations. Mouse tibias and femurs were collected and measured with calipers at the end of the study.

### Treatment of Fgfr3^Y367C/+^ mice.

*Tg-CMV^Cre/+^/Fgfr3^Y367C/+^* mice were tattooed and assigned to treatment groups within each litter at 1 day of age (wild-type mice, *Fgfr3^Y367/+^* mice treated with vehicle, or *Fgfr3^Y367/+^* mice treated with 1.2 mg/kg TYRA-300). TYRA-300 was dissolved in aqueous HCl (3.5 mM) containing 5% dimethyl sulfoxide (DMSO) and administered subcutaneously to *Fgfr3^Y367/+^* mice (*n* = 8) for 15 days. The vehicle was administered to wild-type (*n* = 10) or *Fgfr3^Y367/+^* mice (*n* = 10) for 15 days. Subcutaneous administration was used for this study rather than oral gavage due to the reduced size of the mice. Clinical observations, nasoanal length and tail length measurements were performed every 3 days, and body weights were collected daily.

### Treatment of Fgfr3^N534K/+^ mice.

*Tg-CMV^Cre/+^/Fgfr3^N534K/+^* mice were tattooed and assigned to treatment groups within each litter at 3 days of age (wild-type mice, *Fgfr3^N534K/+^* mice treated with vehicle, or *Fgfr3^N534K/+^* mice treated with 1.8 mg/kg TYRA-300). TYRA-300 was dissolved in aqueous HCl (3.5 mM) containing 5% DMSO and administered subcutaneously to *Fgfr3^N534K/+^* mice (*n* = 9) for 21 days. The vehicle was administered to wild-type (*n* = 12) or *Fgfr3^N534K/+^* mice (*n* = 11) for 21 days. Subcutaneous administration was used for this study rather than oral gavage due to the size of the mice. Clinical observations, nasoanal length, and tail length measurements were performed every 3 days, and body weights were collected daily.

### Bone measurement and x-ray imaging.

Whole body x-ray images were taken of all animals by Faxitron specimen radiography MX-20 (Faxitron X-Ray LLC) following terminal sacrifice. The length of the L4–L6 lumbar vertebra segment was also measured for each animal using calipers, and the height of the L5 vertebral body was measured from Alcian blue/Sirius red images. Long bones and the skull were then excised, and their dimensions were measured using calipers (VWRi819-0013, VWR International). The transverse and sagittal diameters of the foramen magnum were also measured using calipers for the *Fgfr3^Y367/+^* model, and measured using the μCT images for the *Fgfr3^N534K/+^* model.

### Immunohistochemistry.

The right femur and lumbar vertebrae segment L4–L6 were collected and fixed in 4% paraformaldehyde, decalcified with ethylenediaminetetraacetic acid (EDTA) 0.5 M (pH 8) for 2 or 3 weeks, and embedded in paraffin. Serial paraffin sections of 5 μm were stained with hematoxylin, eosin, and Safranin O. For immunohistochemistry, sections were labeled with antibodies against collagen type X (BIOCYC ref. 1-CO097-05-RUO, 1:100 dilution), collagen type I (Novotec, 20151, dilution 1:1,000), pERK (Cell Signaling, 4370S, dilution 1:1,000), and PCNA (Abcam, GR3195972-6, 1:500 dilution) using the Dako Envision Kit. Images were captured with an Olympus PD70-IX2-UCB microscope and CellSens software (Olympus Life Science Solutions). Cells were counted using ImageJ software (NIH, Laboratory for Optical Computational Instrumentation).

### Morphometric analysis of skulls by μCT imaging.

The heads of all mice were isolated, and skin was stripped before being placed in 70% ethanol at 4°C. μCT images of the skulls were measured on the Skyscan-1272 (Bruker) with the following parameters: 10 μm resolution, 680 ms exposure, 70 kV, 142 μA, and 0.350° rotation step. The μCT data were analyzed using DataViewer and CTAn software (version 1.20.8) to perform postprocessing techniques in 3D. The nasio-occipital length and anteroposterior length of the skull were measured using 2D μCT images.

### μCT analyses and imaging of long bones.

Femurs were placed in 70% ethanol at 4°C and measured on the Skyscan-1272 (Bruker) with the following parameters: 60 kV, 166 μA, 0.25 mm aluminum filter. Within the femurs, the trabecular parameters were obtained at 5 μm voxel resolution in a ROI (normalized to 7% of the femur length in size) beginning 255 μm proximal to the distal growth plate for controls, 25 μm for *Fgfr3^Y367C/+^* + vehicle, and 35 μm for *Fgfr3^Y367C/+^* + TYRA-300. The BMD and BV/TV of the metaphysis were measured using on a set of sections located within the secondary spongiosa under the growth plate. The measured volume was chosen to be proportional to the femur length (typically 630 μm for control animals and 310 μm for *Fgfr3^Y367C/+^* + vehicle and 415 μm for *Fgfr3^Y367C/+^* + TYRA-300.) Scan reconstruction was done using NRecon (v 1.7.4.6). Scan orientation was done using DataViewer (v 1.7.0.1). 3D Analysis for trabecular bone was performed using CT Analyzer (v 1.23.0.2).

### Statistics.

Data in graphs are presented as mean ± SEM. Differences between experimental groups measured over time were assessed using a Mann Whitney *U* test at each time point with correction for multiple comparisons using a false discovery rate of 1%. Differences between experimental groups at endpoint were assessed using a Kruskal-Wallis test with Dunn’s multiple comparisons test. The significance threshold was set at *P* < 0.05. Statistical analyses were performed using GraphPad PRISM (v5.03).

### Study approval.

For the wild-type mouse experiments, all animal procedures were approved by the Institutional Animal Care and Use Committee (Charles River CRADL, EB21-011-001) and in agreement with the NIH *Guide for the Care and Use of Laboratory Animals* (National Academies Press, 2011). For the *Fgfr3^Y367C/+^* and *Fgfr3^N534K/+^* mouse experiments, animal procedures were approved by the French Ministry of Research and the Animal Care and Use Committee at Université Paris Cité (APAFIS#24826-2018080216094268 v5) and were conducted in compliance with ethical principles at the Laboratoire d’Expérimentation Animale et de Transgénèse (LEAT) Facility (Imagine Institute, Paris, France).

### Data availability.

All data associated with this study are included in the Supporting Data Values file or are available upon request.

## Author contributions

LLM, RVS, TH, and JHS conceptualized and designed the studies. LLM, RVS, JHS, ELA, CF, and MG developed the methodology. JHS, CL, MG, NK, CF, MN, EAP, MP, QY, and MC performed the experiments. JHS, RVS, MBB, and LLM wrote the manuscript, and LLM, RVS, MBB, GB, RLH, TH, and JHS revised the drafts and reviewed the manuscript.

## Supplementary Material

Supporting data values

## Version 1 04/03/2025

In-Press Preview

## Version 2 05/08/2025

Electronic publication
